# A qualitative study to explore student learning and development of interprofessional collaboration during an online interprofessional education intervention

**DOI:** 10.1186/s12909-023-04885-y

**Published:** 2023-12-14

**Authors:** Hailah Almoghirah, Jan Illing, Hamde Nazar

**Affiliations:** 1 College of Pharmacy, King Saud University, Riyadh, Saudi Arabia; 2 Health Professions Education Centre, RCSI University of Medicine and Health Sciences, Dublin, Ireland; 3 Newcastle University, Newcastle-Upon-Tyne, UK; 4 School of Pharmacy, Newcastle University, Newcastle-Upon-Tyne, UK

**Keywords:** Interprofessional education, Collaboration, Interprofessional professionalism, Entrustable professional activities, Qualitative study

## Abstract

Interprofessional education (IPE) during undergraduate education and training has been found to improve collaboration between health care students. This supports interprofessional working in clinical practice to enhance patient safety and care delivery.

Undergraduate students from pharmacy and medical programmes worked online in pairs to review notes of hospital patients due to be discharged. Students were tasked to complete a discharge letter and undertake an online consultation with a simulated patient prior to discharge. Online interactions were recorded and assessed using a validated tool to measure interprofessional professionalism. Students undertook this intervention in different pairings with different patient cases for three iterations after receiving feedback and undertaking a reflective exercise.

The aim was to investigate the student learning and development that could be used to inform intervention optimisation and scale-up.

Qualitative data were collected from different sources. Method triangulation was employed to develop a comprehensive understanding of the student learning and development. Data was collected from written feedback provided by the assessment team, student reflections on their performance, and from semi-structured interviews conducted with the student pairs and one to one with the assessment team. Content and thematic analysis was used to analyse these data and the Kirkpatrick/Barr evaluation model provided a framework to organise the themes.

Eighteen students (nine from each professional programme) completed the study and a total of 27 IPE sessions were conducted. The assessment team completed 54 assessment tools and 31 student reflections were received (from a maximum of 36). Students were interviewed in their interprofessional pairs to yield nine interview transcripts and one interview was conducted with the assessment team.

Students reported and were observed to improve in interprofessional collaboration over the three iterations following feedback and rehearsal opportunities. Longitudinal observation and assessment of student interprofessional working in changing teams provided the opportunity to capture the influence of interdependence on student performance and assessment of competence.

**Supplementary Information:**

The online version contains supplementary material available at 10.1186/s12909-023-04885-y.

## Introduction

It is well established that teamworking and good communication between healthcare professionals contributes to positive patient outcomes, improved patient safety and job satisfaction within the healthcare workforce [1–3]. In particular, collaborative doctor-pharmacist team-based care has been found to have a positive effect on the management of chronic disease and has reduced the occurrence of medical errors during the transition from hospital in-patient back to primary care [3, 4]. However, there are many reported barriers to working collaboratively, for example existing or perceived hierarchical structures; complexity of healthcare systems and procedures, and silo working [5, 6].

Interprofessional education (IPE) within undergraduate and professional training has been identified as a strategy to help improve teamworking and collaborative communication between different healthcare professions [7].

Educators are challenged to design and create educational and training experiences that provide learners the opportunity to collaborate meaningfully and develop knowledge, skills, and behaviours to support collaborative practice [8, 9]. There is significant IPE scholarship and research which advocate for authentic learning experiences with a high level of fidelity, delivered as a longitudinal programme of experiences, rather than ad hoc isolated sessions with provision of feedback for learners [9, 10]. These are most likely to lead to a sustained impact on the developing skills and behaviours that support later effective working in interprofessional teams.

In this study, we aim to investigate if and how an IPE intervention, facilitated students to become more collaborative. Undergraduate medical and pharmacy students participated in an online IPE intervention that focused on the transition of care from hospital in-patient to primary care. The framework of entrustable professional activities (EPAs) informed the design of this intervention as hospital discharge planning is an identified clinical activity likely to require interprofessional collaboration. Researchers have contested the use of EPAs, which are about entrustment of individuals based on their competence, within the field of IPE, which is about interprofessional collaboration. However, there is recognition that some EPAs are dependent upon interprofessional working and authors acknowledge that assessing interprofessional collaborative competence (reflecting team competencies) is important when assessing EPAs and making entrustment decisions (a unit of professional practice that can be fully entrusted to a trainee, once he or she has demonstrated the necessary competence to execute this activity unsupervised) [11]. The students in this study were assessed and provided with feedback on their individual performance and had the opportunity to undertake the intervention two more times with other students and using further patient cases to apply their learning into practice. In our previous work, we showed that student interprofessional behaviour statistically improved over the three iterations [12]. In this work, we provide greater insight into the ‘*how*’ and ‘*why*’ students developed through this longitudinal experience which could facilitate how educators reconcile using the individually focussed EPAs in the team-based context of IPE.

## Method

Our qualitative study adopted method triangulation of data from a range of sources to investigate in-depth student learning and development. The Consolidated criteria for Reporting Qualitative research Checklist (COREQ) has been used in the reporting of this study [13]. The completed checklist is included in the Supplementary information.

### Participants

A detailed description of the intervention is included in our previous work; [12] however, we provide a brief outline here. Undergraduate students from year five (final year) of the Bachelor of Medicine and Bachelor of Surgery (MBBS) and years three and four (final two years) of the Master of Pharmacy (MPharm) programmes were invited by email through the course coordinators to participate in this study. These students were identified to already have knowledge about the discharge process and previous experience with IPE. The EPA framework was used to design the session and collaborative activities for students [14–16]. Real clinical notes of patients due to be discharged from a local hospital were sourced and anonymised by a clinical pharmacist from the hospital briefed about the intervention. Participants were tasked to meet online (using Zoom: A spatial data visualization tool. (Version 2.0.6) [17] to collaboratively review the patient notes, prepare a patient discharge letter and then undertake a patient consultation to counsel the simulated patient prior to discharge.

Each student was required to complete three IPE sessions 2–3 weeks apart, each taking one hour and were paired with a different student and were provided with a new patient case. Thus, mimicking the transient nature of team working in healthcare. Students were provided with an instructional video at the beginning to describe the educational intervention and asked to complete the consent form to participate as research participants. One day before each scheduled online session, students were provided with the patient case and a template for the discharge letter. During the online session, one medical student and one pharmacy student worked collaboratively to review and discuss the patient notes and prepare a discharge letter. The students were then joined by the simulated patient to undertake a consultation about their hospital discharge. The sessions were recorded with consent and later used for assessing student performance (as described in our previous work) by an assessment team (an academic pharmacist and a general practitioner).

### Data collection

Student behavioural performance was captured using a validated tool, the Interprofessional Professionalism Assessment tool (IPA) which consists of six domains: communication, respect, altruism and caring, excellence, ethics and accountability (covering 26 items) [18]. Assessors also provided written feedback against these domains. These qualitative comments from the IPA tool were collected and matched with each student across the IPE iterations.

Students were provided with their respective IPA scores and comments via the lead research (AH) after each IPE session and asked to reflect on their performance and future learning objectives (*What did you do well? What areas did you find challenging? What do you plan to improve?).* The written answers to these three questions were also collected, and again matched with each student across the IPE iterations. There are other studies which have successfully analysed student reflections following interprofessional educational interventions. This has been done to investigate student learning from such experiences [19–24].

After completing all three IPE sessions, students were involved in an interview to explore their perceptions of the intervention and their learning and development. Interviews were conducted following the last IPE session, so included student pairs.

Lastly, the assessors (*n* = 2) who reviewed the recorded student performance and completed the IPA tool were interviewed together to explore their perceptions and experiences of the intervention, mode of assessment and observed student learning and development. Assessors were provided with information about the study and asked to provide oral consent before participating in the interview.

One assessor was an academic pharmacist with over ten years of educational experience with specific health service research interest in improving hospital to home discharge care; the other assessor was a practicing general practitioner with over five years of educational experience and ten years of clinical practice.

**Fig. 1:**
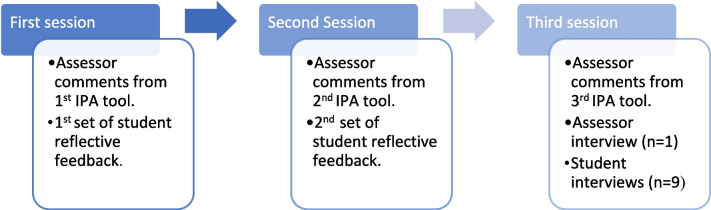
Sources of qualitative data after each IPE session

Interview schedules are included in the Supplementary information. All interviews were audio recorded with consent and transcribed verbatim. The sources of qualitative data after each IPE session illustrated in Fig. 1.

Figure 1 illustrates the sources of qualitative data after each IPE session.

### Data analysis

The data were managed and coded using NVivo 1.2 (426) (QSR International). Manifest content analysis was used to analyse the IPA qualitative comments and the student reflections [25]. Thematic analysis was used to analyse interview data using Braun & Clarke’s, six phases: familiarisation, data coding, generating initial themes, developing, and reviewing themes, refining, defining, and naming themes and writing matters for analysis. A combination of inductive and deductive approaches where used [26]. Initially, inductive coding facilitated the generation of themes from identified codes. The Kirkpatrick/Barr evaluation model [27] was then used as a conceptual framework to help organise the themes. The initial coding was conducted by HA then shared, discussed, and reviewed with the wider research team.

Once all data was analysed, the researcher iteratively compared across the data, particularly data from the different sources across the same student and across the three iterations, to explore convergence, complementarity, and dissonance. The researcher aimed to develop a comprehensive understanding of individual student journeys and of the collective. This approach is also recognised to increase the credibility and dependability of the findings and interpretations [28].

To improve the trustworthiness of the analysis and interpretation, different approaches have been used [29]. During the intervention, we documented all steps. We undertook iterative data collection from different viewpoints. Then, the data were iteratively analysed, and the analysis was shared and discussed with the research team. Member checking was conducted with one of the assessors to clarify some points in the interview and increase understanding prior to analysis. In our previous work, [12] we included a comprehensive description of the IPE intervention and assessment. We have used the COREQ checklist for reporting the study and provided a detailed description about the methods of data collection, data analysis.

### Ethical approval

Ethical approved was obtained from the University Ethics Committee at Newcastle University before starting the study (reference number: 5299/2020) and written consent was obtained from all participants before data collection.

## Results

Eighteen students completed this study: nine medical students and nine pharmacy students. A total of 27 IPE sessions (nine pairs of students completing three IPE sessions) were completed and the assessment team completed 54 IPA assessments across the three IPE iterations. We received 31 reflections from the students while three students failed to send their feedback on five occasions. Nine interviews were conducted with participants in pairs and one interview was conducted with the assessment team.

The themes from the data were organised using the Kirkpatrick/Barr evaluation model, which are: reaction to the intervention; modification of attitudes and perceptions; behavioural change, and benefit to patient. Data from this study did not map to the domain of changing organisational practice.

The themes are presented below with illustrative quotes. We depict whether the quote originated from a medical or pharmacy student or from an assessor (M: medical student (*n* = 9), P: pharmacy student (*n* = 9), A: assessor (*n* = 2)), and from which data source (I: interview (*n* = 9, one per student pair), R: student reflection (*n* = 2 per student), IPA (*n* = 3 per student): comment included in the IPA tool).

### Reaction to the intervention

After the first sessions, some students felt challenged and expressed feelings of struggling to work with someone else for first time.

"*I think it’s just the first time, I've not really done anything like this before. So, everything was new, I didn’t know what my role was. I didn’t know what to expect of the pharmacy student"* (M6, I).

After receiving the assessor feedback and then repeating the session, reactions improved as students expressed that they felt that they knew what to do and they felt that the session activities were closely linked to their future practice.

*"We worked together well despite not knowing each other which was very representative of real-life situations in the world of work*" (P8, R1).

After the third session, student awareness about the importance of interprofessional working improved and they became more engaged with the other professions' perspective. Also, students enjoyed the experience and found it useful for their education.

*" I struggled to understand what is the role of other healthcare professionals in this particular scenario. But…with three sessions I progressively saw it more and more useful and figured out how better to work with our Pharmacy colleague"* (M3, I).

The assessors also acknowledged that these IPE activities related well to real practice and the tasks were appropriate for students to work together to deliver safe and effective care.

*" So, I really like the authenticity of that. The next thing was they were doing the activity, which they would actually have to do in practice. So again, the authenticity is really high"* (A2, I).

### Modification of attitudes and perceptions

After the first sessions, teamwork was reported as an issue with many of the students. Some found themselves doing all the work without involving their colleagues, while other students relied on their colleague to do the bulk of the activity.

*" I felt that I took on the lead role with the discussion with the patient at the end"* (M7, R1).

*"Potentially taking some time to get to know other colleagues in a professional sense may help more shy or passive personalities engage more"* (P4, IPA1).

After the second sessions, students began to be more positive about their experience of teamworking and they became more comfortable and confident when working with each other. However, for some the role of each other was still not completely clear and some were struggling to contribute more assertively in the session.

*"Easy collaborative approach demonstrating initiative and confidence in contribution"* (P3, IPA2).

*"I feel there was better communication between me and the medic and I felt more comfortable with them"* (P4, R2).

"*It was also a bit confusing as to which part shall I talk about and which part should the pharmacist talk about* [in the patient consultation]*"* (M9, R2).

On completing the third sessions, student attitudes were improved when working with their colleagues such as respecting each other, becoming more confident and comfortable, giving each other space, asking each other questions, etc. As a result, students felt that these attitudes led to better teamwork.

*"We needed time to figure out what’s going on and get more confident with what we are doing. And therefore, we ended up working as a better team…as the sessions progressed"* (M3, I).

*" No significant areas for improvement. Generally good collaborative approach"* (P8, IPA3).

The assessors also mentioned the positive attitudes demonstrated by students during the sessions which contributed to teamwork such as asking each other questions and clarifying each other’s roles. Assessors felt that providing feedback to students about their performance was an important facilitator in attitude improvement.

*"I think the fact that they were able to get feedback after each of the iterations were really helpful because they could see the comments around whether they talked enough or didn't talk enough. Or weren't proactive enough… how they manage the patient. And when you saw their reflective answers to the questions they were asked, you could see that they'd actually consider that, that feedback. And then when you saw the next time, it was really interesting to see how they had or hadn't taken that on board and changed and adapted"* (A1, I).

### Acquisition of knowledge and skills

Initially, students grappled to understand the role of each other in the context of the IPE tasks.

*" I guess in the first session that was a challenge of understanding our roles as practitioners and what to expect from one another"* (M6, I).

*" Spending a moment to verify each other’s roles and potential contribution to this scenario would have been helpful in ensuring an organized approach to the task"* (M2, IPA1).

As the sessions progressed, students gained this understanding which facilitated more collaborative dynamics.

*"Worked better with pharmacist as I understood their role and my role in completing the task"* (M1, R2).

*"Very open to learn from others and this sets good foundation for interprofessional working"* (P6, IPA2).

On completing the third sessions, students not only agreed about learning more about each other’s roles but also about the importance of each profession in providing safe and effective care for patients.

*"… so it’s not this hierarchy that people think about medical doctors, you can’t speak to pharmacy students, we have to work together. I think learning how important it is to have two professions, that really brought it out, this experience helped me see this"* (P9, I).

The assessors supported this in their reported observations of students asking each other about their roles and responsibilities and how this enabled more open communication.

“*I think they also learned about a non-hierarchical type of communication style, you know that both parties could contribute to the situation"* (A1, I).

### Behavioural change

Some students initially demonstrated good communication and teamwork, whilst some students struggled to work effectively in a team. This was exemplified where some students mentioned that they did not manage the tasks well, with no clear delineation of roles.

*"Unfortunately, the pharmacist had to complete the discharge letter whilst I talked to the patient rather than us both doing it together"* (M7, R1).

*"…, it would have been safer to briefly plan how to manage the consultation since you are still quite unfamiliar with each other and each other’s potential approach to consultations*" (P3, IPA1).

However, by the second sessions, student awareness appeared to improve. Students mentioned some strategies that helped collaboration, such as giving each other a chance and space to talk, becoming clearer in their communication, and behaving more confidently with each other.

*"I demonstrated confidence and gave equal input as the other student"* (P7, R2).

*"Open, relaxed and engaging approach to communication"* (M4, IPA2).

By the third sessions, students described their behaviours with positivity.

*"We were able to communicate effectively and most of the times we'd agree, but when we don’t, we resolved it quite quickly on which way to go with it" (*P6, I).

*"Made valuable contributions where appropriate, and was not overly verbose. So gave the appearance of quiet confidence which is reassuring"* (P8, IPA3).

Students explained more techniques to collaborate with their colleagues, such as trusting each other, allowing each other a chance to contribute and splitting of responsibilities.

*"I think, from the feedback I received, it was a relaxed environment with me and the medical student and it said that there was good interaction between us and good teamwork and collaboration. There wasn’t like silence all the time"* (P9, I).

The assessors observed student behaviours generally improved across the IPE sessions. They described that the feedback on performance would have facilitated this as students were provided with some insight about how they behaved so that they could consider this and work to improve. However, the assessors mentioned that some students continued to struggle with some behaviour such as body language despite feedback and repetition. They opined that maybe this could have been due to personal characteristics, the influence of the person they were working with, the difficulty of the patient cases or they simply they didn’t understand the feedback provided.

*"So globally there was improvement"* (A1, I).

*"Despite they improved, you could tell that the person they've worked with impacted their performance. So maybe they could have improved further if the person they're working with was more positive."* (A1, I).

### Benefit to patient

For this theme, the student self-perception about patient experience and assessors' comments were used as proxy measures.

After the first sessions, students were positive about their communication with patients, and they felt that they dealt with the patient expressed need and provided the required care.

*"Talked with the patient well and patient felt happy with description of care to be provided *" (M6, R1).

*"Person-centred with clear patient consultation*" (P9, IPA1).

After the second sessions, students continued to feel positive with the patient outcome where they had demonstrated person-centred care, communicated clearly, and provided a comprehensive consultation. However, some students mentioned struggling to answer patient questions.

*"We collaborated and focused on the patient’s needs and concerns"* (P7, R2).

*"I think that I struggled most to answer patient questions in which I was not confident in the answer. I found myself responding that I didn't know or would find out, which I was worried might look unprofessional and not satisfy some patients"* (M4, R2).

After the third sessions, students used and mentioned a number of techniques they found useful to help ensure a positive experience for the patient such as creating a relaxed environment during consultation, demonstrating empathy, giving space to ask questions, listening to their concerns, trying to be professional, not overloading the patient with information and trying to understand the patient perspective.

*"Was empathetic with the patient, considered her particular concerns and responded reassuringly"* (P7, IPA3).

*"I think the patient in all three cases left quite happy and they’d had all their questions answered, so I feel that we communicated well with them and with each other"* (P3, I).

The assessors agreed that students were professional and that there was harmonisation between students when conducting the consultation which reflected interprofessional collaboration.

*"There was an element of synchronising who was going to say what; they had to both, you know, come across as really professional and negotiate how the patient was responding"* (A2, I).

Further details and quotations are provided in the Supplementary information.

For some students, three iterations of the interprofessional education session led to a positive change in their teamworking and communication. In Table 1, we illustrate a journey of two students who demonstrated positive development across three iterations. In this example, the medical student (M7) initially demonstrated a dominant and verbose personality and approach, which was perceived as overly assertive by their pharmacist counterpart (P7). Conversely, the pharmacy student (P7) portrayed a passive demeanour at the outset. However, over the three sessions, we observe M7 developing a more considerate and inclusive communication approach and P7 becoming more assertive and engaging. This is reflected both in their respective partner’s perception of the interaction and the comments provided by the assessment team.

**Table 1:** Examples of student change over three iterations

	**Student reflection on their performance**	**Student's partner observation on their performance**	**Assessor comments about student performance**
**First example (M7)**			
**1**^**st**^** session (M7 and P7)**	*" I felt that I took on the lead role with the discussion with the patient at the end. This was not my intention as I wanted it to be a more open discussion between the 3 of us but inadvertently was drawn into it"* (M7, R1) *"I Will attempt to allow the other HCP to take the lead/share the decision and discussion at the end"* (M7, R1)	*" Due to my lack of experience with discharge letters and my shy personality, I did not contribute as much as I would have liked to"* (P7,R1) *" I observed and let the other student guide me through the session and in doing this, learnt a lot"* (P7, R1) *" I wanted to speak during the consultation however I felt rude butting in when the other student was speaking and didn't get much of an opportunity to speak."* (P7,R1)	*"Quite verbose at times which could be perceived as somewhat overwhelming. A quieter personality may struggle to feel they would be heard or could contribute"* (IPA1,M7)
**2**^**nd**^** session (M7 and P2)**	*We worked well as a team and had a clear agenda for the consultation with the patient" (*M7, R2) *"Clear communication with the pharmacist regarding important medication changes, particularly in the context of the case"* (M7, R2)	*"I thought that was quite nice like there was a lot of collaboration"* (P2, I)	*"More restrained communication than previous session. Gave space for MPharm student to ask questions, clarify and pose suggestions"* (IPA2, M7)
**3**^**rd**^** session (M7 and P9)**	*"I think I got a lot better at the shared communication aspect, particularly in the first one" (*M7, I) *"Have a more dynamic approach to it and address each other's issues, rather than inadvertently do what I did, which is the worst thing you could possibly do, and just go in and take control, which is a horrendous way to do it. Never do that, ever. "* (M7, I)	*" but I think it was hard for me to give my opinion because they were talking a lot so I felt rude to interrupt them but I think they realised"* (P9, I)	*"Worked well with MPharm student with a very friendly and easy approach. There was a good discussion between the two students as they considered each other’s opinions and contributions"* (IPA3, M7)
**Second Example (P7)**			
**1**^**st**^** session (P7 and M7)**	*"Due to my lack of experience with discharge letters and my shy personality, I did not contribute as much as I would have liked to, especially during the consultation"* (P7, R1) *"I plan to contribute more to the session and ensure I have time to speak to the patient during the consultation. I would like to come across more confident"* (P7, R1)	*"I felt that I took on the lead role with the discussion with the patient at the end. This was not my intention as I wanted it to be a more open discussion between the 3 of us but inadvertently was drawn into it"* (M7, R1)	*"The MPharm student did not contribute to the patient consultation, which is a shame because it gave the impression of professional boundaries/hierarchies"* (IPA1, P7)
**2**^**nd**^** session (P7 and M2)**	*"I demonstrated confidence and gave equal input as the other student"* (P7, R2) *" I find it challenging to be assertive, it was hard for me to explain the importance of medication adherence to the patient, however I think I managed this well"* (P7, R2)	*" I worked well with the MPharm student in this session and clarified more about her background and what she wanted to cover in our discussion than I had in the first session"* (M2, R2)	*" Worked well with MBBS student. Easy flow of conversation and management of the case. Assertive and directive in managing the case"* (IPA2, P7)
**3**^**rd**^** session** **(P7 and M6)**	*"I think what went well was just being open with working with each other, and not being stuck in your own ways, being open to change and looking at how you can improve more as a team, rather than individually"* (P7, I)	*" Together we can provide a really balanced discussion with the patient and reassure them on a safe discharge"* (M6, I)	*" Engaged and listened well to the MBBS student and shared thoughts and perspectives easily. Was open to discuss how to manage the patient consultation. Was warm with the patient and responded appropriately to the patient concerns"* (IPA3, P7)

## Discussion

This study demonstrated that interprofessional working can improve with three iterations following feedback and further rehearsal opportunities. In our previous work, we were able to demonstrate a statistically significant improvement with rehearsal, while this study, illustrated evidence of improved collaboration [12].

The iterative nature of the intervention with feedback enabled students to reflect on their performance and make further adjustments so that they were supported to become more self-aware and purposefully develop. This observation is supported by Kolb's Experiential learning theory [30] where students learn by experience, reflection, and further development in a cyclical way. Reflective practice in interprofessional education interventions is not new. Positive experiences using reflective practice in pharmacy and other health care professions has been published previously in the literature [21–24]. These studies showed that reflective practice did faciltate students towardss continuous improvement and skills development [21–24].

Providing students with personal feedback enables feedforward as advised in literature [31–33]. In the main, previous IPE interventions have been a one-off session or delivered in a longitudinal manner and the students were assessed either using pre/post or just post assessment [34–40]. Students in these studies did not receive feedback nor were they facilitated to reflect on their performance and given another opportunity to put the feedback into practice as in our intervention.

The authentic learning experience with a simulated patient and adopting the premise of EPAs was positively received and our multi-assessment approach allowed us to successfully capture the impact of the IPE intervention across most of Kirkpatrick/Barr evaluation levels. Other similar studies have generally focused on assessing if the IPE activities impacted learners across one or two specific levels of Kirkpatrick/Barr evaluation model; either learner’s reaction, modification of attitudes and perceptions, behavioural change, or patient benefit [34–40].

The opportunity to observe student performance with different student partners has highlighted how behaviours and approaches of a student can influence how their counterpart then behaves and performs. This is otherwise known as interdependence: "*patterns of interaction between individuals, working collaboratively, that can afford or constrain one's performance and potentially shape the practice of a broader healthcare team*" [41] (p2). It is important for educators to consider interdependence during student assessment, especially in the context of EPAs that are inherently dependent upon interprofessional working. Some IPE assessment tools identified in literature have some items that try to capture the nature of interdependence, whilst some tools offer a space for qualitative comments such as textual feedback [42]. In our study, the assessors recorded qualitative comments in the IPA tool to describe the dynamics between the students and observations about its impact on performance. To our knowledge, this is the first IPE study which has required students to work collaboratively on the same task with different colleagues and has enabled the impact of interdependence to be clearly demonstrated.

Our study is limited by the small number of participants, recruited from just two programmes from one institution. It is possible that these students were more motivated than other students and the pharmacy tutor was known to the students, hence scaling up to a full cohort is important. However, we have compensated by capturing, analysing, and triangulating data from different sources to best investigate our research question. The student interviews were conducted in pairs. This could have meant that potential power dynamics may have impacted how the students responded and interacted within the interview. As with many educational interventional studies, a longer period to assess the sustained impact of the intervention would be invaluable [43]. However, our study findings strongly suggest that any endeavour to develop interprofessional collaboration between undergraduate healthcare students is best delivered iteratively with opportunity for feedback and reflection. This study also lacks the patient perspective on student interprofessional collaboration which could represent another patient outcome.

Our intervention was focussed on the discharge process, but the approach of conducting this intervention could be generalised to other scenarios where interprofessional working might be expected, *e.g.,* care planning, solving patient safety issues. From our work, we would recommend that educators aiming to design and deliver IPE interventions with an assessment should consider the following strategies: framing their IPE intervention around EPA's that depend upon interprofessional working; providing multiple opportunities for the students to complete it with different peers and provide multiple opportunities for the students to get feedback and engage in reflection.

## Conclusion

Interprofessional collaboration improved after three iterations following individual feedback and further rehearsal opportunities. Having a quantitative measure is important to assess the student's performance but having the opportunity to provide qualitative feedback is also important to gain greater understanding of student performance and capturing the impact of interdependence. Future work could assess this educational and assessment approach in other IPE environments or scenarios on a larger scale. Also, collecting feedback from the patient and peer would provide further perspectives about students' interprofessional collaboration.

### Supplementary Information

**Additional file 1.**

## Data Availability

The datasets used and/or analysed during the current study are available from the corresponding author on reasonable request.
