# Supplementary material for: A qualitative study to explore student learning and development of interprofessional collaboration during an online interprofessional education intervention
Source: BMC Med Educ. 2023 Dec 14;23:957. doi: 10.1186/s12909-023-04885-y (PMC10720163; doi:10.1186/s12909-023-04885-y)
Supplement: Supplementary file 1 — Additional file 1. [file 12909_2023_4885_MOESM1_ESM.zip › Supplementary Materials.pdf]

Supplementation information

S1: Consolidated criteria for Reporting Qualitative research (COREQ) checklist
S2: Further details for COREQ
S3: Interview Guide
S4: Assessor interview questions
S5: Interpretation of different qualitative data using Kirkpatrick evaluation levels.
S6: Illustrative quotes from different data presented using Kirkpatrick evaluation model.

<u>S1:</u> The Consolidated criteria for Reporting Qualitative research (COREQ) checklist:

Checklist of items that should be included in reports of qualitative studies.

Please see attached PDF document

<u>S2:</u> Further details for COREQ:

Personal characteristic:

- HA conducted the focus group using a semi structure guide. HA is a female having a master's degree in clinical pharmacy and work as a demonstrator in clinical pharmacy department at King Saud university, SA since 2011. She was teaching pre-licensure pharmacy students different coursed under clinical pharmacy such as clinical skills, pharmaceutical care, and ethics. Currently, HA is a PhD student at Newcastle University focus on studying interprofessional education at pre-licensure level between health care students. Recently, she received the following training:
  - Analysis of qualitative data – 3day course.
  - NVivo training - 1day course.
  - Qualitative methods sessions as a part of Fundamentals of Research module – 4sessions.

Relationship with participants:

No relationship was established prior to study commencement and none of the participants knew the interviewer. The researcher is academic pharmacist who teach students different subjects under clinical skills and pharmaceutical care. Researcher was involved in clinical rotations during Master degree. During discharge of patient counselling from different professions is a required and those people need to work together collaboratively to ensure that patient gets information that they need.

Theoretical framework:

Kolb's Experiential Learning Theory used as a theoretical underpinning for Interprofessional Education and Thematic framework analysis was used that is underpinned by adapted grounded theory.

Data collection

Interview guide and questions were created by the authors and no pilot testing was undertaken. No repeat interview was undertaken

Field notes was used after each reflection feedback and interview. The length of the interview varied between 12minutes to 36 minutes. Transcripts were not returned to participants for comment and/or correction.

S3: Interview Guide:

Interview guide

Firstly, I would like to audio record our interview so I can go over it later and type it up. I promise to destroy the recording after I have completed this study; would that be okay?
I am speaking with …….., and ……..

Thank you for agreeing to participate in this study which aims to investigate students' performance as they engaged in an interprofessional education learning using an evidence-based assessment approach. I would be grateful if you could give me a verbal confirmation that you have seen and read the information sheet and that you have provide oral consent to undertake this discussion.

This discussion will take about 15 minutes to 30 minutes. I am interested to hear about your experience, how such type of education may impact in your performance and effect your communication, caring, ethics and accountability with different members of the team. All information is confidential. It will not be possible to link the information you disclose back to you. Participation is entirely voluntary.

Do you have any questions before we start?

Questions for students:

- Can we start by asking about your views of this IPE experience?
- What if anything do you think you learned?
- What did you learn about the other profession you were working with?
- What do you think went well?
- What were the challenges involved?
- What did you gain from repeating the intervention?
- What did you learn from the patient?
- Do you have any suggestions on how this could be improved in future?

Thank you for agreeing to participate in this focus group. It has been very useful for me to understand your views of how your performance have been changed after enrolment in interprofessional work. Before we finish, would you like to tell us anything else about your experience? Or any other comments you may have forgotten to add during the discussion?

Thanks again for making the time to participate in the focus group. If you have any concerns or require further information, you can contact me as the primary researcher, or the other contacts provided on the participant information sheet. Let me know if you require these contacts to be resent to you.

S4: Assessor interview questions:

- What are your views of the IPE experience?
- What do you think the students learned from each other by doing this activity?
- Did you see any improvement in the students over time?
- How did you find using the IPA tool?
- Any other comments about the intervention?
- Do you have any suggestions on how this could be improved in future?

<u>S5:</u> Interpretation of different qualitative data using Kirkpatrick evaluation levels.

		Data set						
		Assessor comments from 1st IPA tools	Student feedback after 1st sessions	Assessor comments from 2nd IPA tools	Student feedback after 2nd sessions	Student feedback from focus group	Assessor feedback form focus group	Assessor comments from 3rd IPA tools
**Kirkpatrick evaluation levels**	**1: Reaction to the intervention**	-	+ Very sketchy at the first session.<br>+ Familiarisation during the first session.<br>+ New thing<br><br>- Nervous to work with someone they have not worked with before.<br>- Didn't know the role of each.	-	+ Knew what to do.<br>+ Represent a real-life situation.	+ Good to practice working with someone for first time.<br>+Understand how to better work with another profession.<br>+ Good for their learning.<br>+ Nice to have a different perspective.<br>+ Each one added his input.	+ Helpful.<br>+ Authentic activity.<br>+ Innovative.	-
	**2a: Modification of attitude and perception**	+ Appreciation of each other.<br>+ Assertive approach<br>+ Collaborative approach.<br>+ Confident approach<br>+ Good interaction with each other.<br>+ Open communication with each other.<br>+ Respectful and professional approach	+ Good relationship with each other.<br>+ Used the time well.<br>+ Covered the case thoroughly.<br>+ Listened more.<br>+ Asked each other for clarification of unknown information.<br>+ Worked collaboratively.<br>+ Engaged well with each other.<br><br>- Challenged to understand how can work in a team.<br>- No teamwork, one student did the job.	+ Assertive when working with each other.<br>+ Calm approach<br>+ Collaborative approach<br>+ Helped each other.<br>+ Open communication style.<br>+ Respectful for each other.<br>+ No significant area for improving interprofessional professionalism.	+ Listened to feedback.<br>+ Improved teamwork.<br>+ Collaborative approach.<br>+ Better communication. + More confident.<br><br>- Not clear on the role of each other.<br>- Challenging to be assertive.	+Working well with each other<br>+ Asked each other about their opinion<br>+ Respected each other's roles.<br>+ A lot of collaboration.<br>+ Communicating well with people.<br>+ More confident.<br>+ More comfortable.<br>+ Improved teamwork<br>+ Gave each other space to talk<br>+ Trusted each other	+ Asked each other a question if they don't know.<br>+ Changed or adapted attitudes after feedback.	+ Assertive.<br>+ Collaborative approach.<br>+ Confident.<br>+ Open communication.<br>+ No significant area for improving interprofessional professionalism.<br><br>-Quite passive.

		- Need to allow other to contribute.<br>- Need to be more assertive.<br>- Need more planning between students.	- All talked at the same time.<br>- Nervous talking to the other students.<br>- Depended on each other.	-Need to be assertive.				
	**2b: Acquisition of Knowledge and skills**	+ Learned from other.<br><br>-Need to understand each other role.	+ Understood each other's roles.<br>+ Know what is important to both professions.<br><br>- Challenge of understanding each other roles.	+ Learned from each other	+ Understood each others' role.	+ Learning more about each others' roles.<br>+ Understanding more how to provide interprofessional care<br>+ Knowing the level of knowledge of each other.<br>+ Learning to ask each other unknown information.<br>+ Communicate with each other better.<br>+ Importance of different professions.<br>+ Different professions can have different priorities.	+ Learned about a non-hierarchical type of communication style.<br>+ Teaching each other different things.	+ Learned from each other.
	**3: Behavioural change**	+ Appreciation of each other.<br>+ Assertive with each other.<br>+ Collaborative approach.<br>+ Confident.<br>+ Good interaction with each other.	+ Communicated well with each other.<br>+ Worked well as a team.<br>+ Good communication.<br><br>- Not clarifying each others' role.	+ Assertive approach<br>+ Calm approach<br>+ Collaborative approach<br>+ Helped one another.	+ Worked better with pharmacist.<br><br>+ Gave colleague more time to speak and contribute.<br>+ Engaged with the other student well.<br>+ Worked well as a team	+ A lot of collaboration.<br>+ Asked about each others' opinion.<br>+ Good interaction.<br>+ Divided the duty<br>+ Good teamwork	+ Generally, student behaviour was improved<br>+/- Mixed behavioural change in some behaviours depending on complexity of the patient case and the person they were working with. (Interdependence)	+ Assertive.<br>+ Collaborative approach.<br>+ Confident.<br>+ Open communication.<br>+ No significant area for improving

		+ Open communication.<br>+ Respectful and professional with each other.<br><br>- Need to allow each other to contribute.<br>- Need to be more assertive.<br>- Need more planning.	- Struggled to allow sufficient time for another colleague to contribute.<br>- No teamwork.	+ Open communication style.<br>+ Respectful for each other.<br>+ No significant area for improving interprofessional professionalism.<br><br>-Need to be assertive.	+ Demonstrated confidence and gave equal input as the other student.<br>+ Clear communication.	+ Communicated effectively	+ Some behaviours improved after feedback and repetition.	interprofessional professionalism.<br>- Quite passive.
	**4b Benefit to patient.**	+ Person centred approach.<br><br>- Need to be more patient-centred<br>- Need to use less jargon.	+ Communicated well.<br>+ Patient was happy.<br>+ Dealt with the medical problems well.<br>+ Good safety netting.	+ Person centred approach	+ Provided patient centred approach.<br>+ Communicating clearly.<br><br>+ Clearer plan for follow-up<br>+ Comprehensive approach to the consultation.<br><br>- Struggled to answer patient questions.	+ Patient could have different perspective.<br>+ Empathy with patient.<br>+ Patient was happy.<br>+ All questions answered.<br>+ Didn't provide too much information.<br>+ Clear information.<br>+ Listened to patient concerns.<br>+ Professional with the patient.<br>+ Provided a comfortable consultation.	+ Professional with patient.<br>+ Synchronising during the consultation.	+ Concise with patient.<br>+ Considerate with patient.<br>+ Person centred approach.<br><br>- Provide a lot of information for patient.<br>- Need to use less jargon.

S6: Illustrative quotes from different data presented using Kirkpatrick evaluation model.

		Assessor comments from 1st IPA tools	Student feedback after 1st sessions	Assessor comments from 2nd IPA tools	Student feedback after 2nd sessions	Student feedback from focus group	Assessor feedback form focus group	Assessor comments from 3rd IPA tools
**Kirkpatrick evaluation levels**	**1: Reaction to the intervention**	-	*"I was nervous for the first session, thinking that it was going to be quite challenging to work with someone I hadn't met over zoom. But then it was actually fine"* (P2, I)<br><br>*" I think it's just the first time, I've not really done anything like this before. So, everything was new, I didn't know what my role was, I didn't know what to expect of the pharmacy student"* (M6, I)	-	*" And then the next two, it was like, right, we are here to do this, or read this, you read this, we'll do together"* (M1, I)<br><br>*" We worked together well despite not knowing each other which was very representative of real life situations in the world of work"* (P8, 2nd iteration)	*"I feel it was good, like, I think it was good to be able to interact with, like, different medical students and I think everyone, like, comes like… we all, like, learnt something. But I think everyone comes, like, a different, sort of like, angle into things"* (P9, I)<br><br>*" I struggle to understand what is the role of other healthcare professionals in this particular scenario. But, kind of like, with three sessions I progressively saw it more and more useful and figured out how better to* …	*" So I really like the authenticity of that. The next thing was they were doing the activity, which they would actually have to do in practice. So again, the authenticity is really high"* (A2,I)<br><br>*"Also, I think it was helpful that it was challenging enough for them to need to work together. So almost drove them to have to, you know, converse with one another. Ask questions, ask for clarification."* (A1,I)	-

						work with our Pharmacy colleague" (M3, I)		
	**2a: Modification of attitude and perception**	*"Potentially taking some time to get to know other colleagues in a professional sense may help more shy or passive personalities engage more"* (IPA1,P4)<br><br>*"Really engaging, open and transparent communication approach"* (IPA1,P3)	*" I felt that I took on the lead role with the discussion with the patient at the end"* (M7,R1)<br><br>*" I think that I communicated well with the MBBS student when I did not understand something on the patient notes and found that we did ultimately work well together"* (P5, R1)	*"Easy collaborative approach demonstrating initiative and confidence in contribution"* (IPA2, P3)<br><br>*"Being better prepared will mean that the student will be more confident and potentially assertive to manage the patient case and consultation"* (IPA2, P9)	*" I think I have managed to listen to feedback and improve my teamworking skills"* (M3, R2)<br><br>*"I feel there was better communication between me and the medic and I felt more comfortable"* (P4, R2<br><br>*"It was also a bit confusing as to which part shall I talk about and which part should the pharmacist talk about"* (M9, R2)<br><br>*"Since I find it challenging to be assertive, it was hard for me to explain the importance of medication adherence to the patient, however I*	*" I think between the first and last session if I watched the recording I would see a difference in how I interacted with the med student and how confident I am talking to the patient because I know I felt a bit more comfortable with it towards the end"* (P2, I)<br><br>*" We needed time to figure out what's going on and get more confident with what we are doing. And therefore, we ended up working as a better team at the, kind of, as the sessions progressed"* (M3, I)	*" I think it was really nice to hear them actually ask really, you know, direct questions. What would a pharmacist do in this situation? Or what does this mean to a doctor? We don't understand this? And is this what would happen in normal practice? Those kind of questions, you know, really prompted the students to ask one another about their roles "* (A2,I)<br><br>*" I think the fact that they were able to get feedback after each of the iterations were really helpful*	*" No significant areas for improvement. Generally good collaborative approach"* (IPA3, P8)<br><br>*"Open approach to communication"* (IPA3, P9)

					think I managed this well" (P17, R2)		*because they could see the comments around whether they talked enough or didn't talk enough. Or weren't proactive enough, or whether they, how they manage the patient. And when you saw their reflective answers to the question they were asked, you could see that they'd actually consider that, that feedback. And then when you saw the next time, it was really interesting to see how they had or hadn't taken that on board and changed and adapted"* (A1,I)	
	**2b: Acquisition of Knowledge and skills**	*" Spending a moment to verify each other's roles and potential*	*" I guess in the first session that was a challenge of understanding*	*"Very open to learn from others and this sets good foundation for interprofessional*	*" I feel like the medical student and I communicated very well with each*	*" I feel like over time I've learnt what I'm good at and what the pharmacy*	*"I think it was really nice to hear them actually ask really, you know,*	-

		contribution to this scenario would have been helpful in ensuring an organized approach to the task" (IPA1, M2)<br><br>*"Also it would help to orientate each other to what each of you might be expected to contribute to this scenario "* (IPA1, P9)	*our roles as practitioners and what to expect from one another"* (M6, I)<br><br>*" flagging what is 'important' to both of us so that we can draw together a picture of the patient and what is required in the discharge"* (M7, R1)	*working"* (IPA2, P5)	*other and had a good understanding of each other's roles"* (P7, R2)<br><br>*" Worked better with pharmacist as I understood their role and my role in completing the task"* (M1, R2)	*students are good at, and we're good at different things and it's really helpful"* (M6, I)<br><br>*"How to communicate with medical students better, and also how to communicate with patients when I'm with a medical student as well. Yeah, just adapting my communication to them"* (P3, I)<br><br>*"I think in general as well how to work well with medical students, so it's not this hierarchy that people think medical doctors you can't speak to pharmacy students, we have to work together. I think learning how important it is to have two*	*direct questions. What would a pharmacist do in this situation? Or what does this mean to a doctor? We don't understand this? And is this what would happen in normal practice? "* (A2, I)<br><br>*"I think they also learned about a non hierarchical type of communication style, you know that both parties could contribute to the situation"* (A1, I)<br><br>*"I thought it was really good opportunity to teach each other different things"* (A2, I)	

						professions, that really brought it out, this experience helped me see this" (P9, I)		
	**3: Behavioural change**	*"Also, even though the patient consultation went very well, it would have been safer to briefly plan how to manage the consultation since you are still quite unfamiliar with each other and each other's potential approach to consultations*" (IPA1, P3)<br><br>*"The student was assertive and confident in managing the scenario and task"* (IPA1, M1)	*"Attempted to complete the discharge letter and communicated well with the other participant"* (M5, R1)<br><br>*"Unfortunately the pharmacist had to complete the discharge letter whilst I talked to the patient rather than us both doing it together"* (M7, R1)	*" Open, relaxed and engaging approach to communication"* (IPA2, M4)<br><br>*"Easy collaborative approach demonstrating initiative and confidence in contribution"* (IPA2, P3)	*"Built on previous experience and completed the task in a much more time efficient manner. Worked better with pharmacist"* (M1, R2)<br><br>*"I demonstrated confidence and gave equal input as the other student. We worked together well despite not knowing each other which was very representative of real life situations in the world of work"* (P6, R2)	*"Because we were much more confident in what we were doing that we worked better together and were more aware of each other's roles I guess"* (M4, I)<br><br>*"I think, from the feedback I received, it was a relaxed environment with me and the medical student and it said that there was good interaction between us and good teamwork and collaboration. There wasn't like silence all the time"* (P9, I)	*"So globally there was improvement"* (A1, I)<br><br>*"Despite the improved you could tell that the person they've worked with impacted their performance. So maybe they could have improved further if the person they're working with was more positive. You know had put more positive interdependence"* (A1,I)	*"Made valuable contributions where appropriate, and was not overly verbose. So gave the appearance of quiet confidence which is reassuring"* (IPA3, P8)<br><br>*"Open, relaxed and engaging approach to communication"* (IPA3, M4)

	**4b Benefit to patient.**	*" Person-centred with clear patient consultation*" (IPA1, P9)<br><br>*"Also considering the patient in this scenario would help keep the consultation non-hierarchical and collaborative"* (IPA1, M1)	*" I also felt that I gave advice within my scope and communicated well about how to manage the patient's pain"* (P5, R1)<br><br>*"Talked with the patient well and patient felt happy with description of care to be provided"* (M6,R1)	*"Very person-centred with clear patient consultation"* (IPA2,M9)	*"We collaborated and focused on the patients needs and concerns"* (P6, R2)<br><br>*"I also feel I had a patient centred approach"* (M2,R2)<br><br>*"I think that I struggled most to answer patient questions in which I was not confident in the answer. I found myself responding that I didn't know or would find out, which I was worried might look unprofessional and not satisfy some patients"* (M4,R2)	"*I think the patient in all three cases left quite happy and they'd had all their questions answered, so I feel that we communicated well with them and with each other"* (P2, I)<br><br>*"I do think we end up with decently written discharge letters. And provide the optimal patient care*" (M3, I)	*"There was an element of synchronising who was going to say what they had to both, you know, come across as really professional and negotiate how the patient was responding"* (A2, I)	*" Was empathetic with the patient, considered her particular concerns and responded reassuringly"* (IPA3, P6)<br><br>*"Consider less jargon and simpler approach to the patient consultation"* (IPA3, P8)
